# Supplementary material for: Optical coherence tomography in soft matter
Source: Soft Matter. 2025 Apr 10;21(18):3425–42. doi: 10.1039/d4sm01537a (PMC12035809; doi:10.1039/d4sm01537a)
Supplement: SM-021-D4SM01537A-s001 [file SM-021-D4SM01537A-s001.pdf]

Supplementary Information (SI) for Soft Matter.
This journal is © The Royal Society of Chemistry 2025

Supplementary Information :

Optical Coherence Tomography in Soft Matter

Kasra Amini[a,‡], Cornelius Wittig[a,‡], Sofia Saoncella[a], Outi Tammisola[b], Fredrik Lundell[a], and Shervin Bagheri[a]

1 softOCT – A Python package

In this tutorial review, we use the Python package *softOCT* to provide the necessary functions and code examples. *softOCT* provides several functions that can be used to extract information from OCT data. It includes functionality for detecting surfaces and interfaces, isolating phases in multiphase problems, and Doppler-OCT velocimetry. These functions assume that the data is supplied in the form of .tiff image stacks or otherwise converted to numpy ndarrays of intensity / phase.

1.1 Example 1 – Simple thresholding

This example is used in Section 3.1.1. An oil droplet is trapped in a milk-filled microchannel. The milk scatters light, whereas the oil appears transparent. This examples shows how to load and binarize OCT intensity measurements, if a clear separation between the foreground and background exists. Finally, the interface between oil and milk is detected and plotted.

1.2 Example 2 – Complex thresholding

This example is used in Section 3.1.2. A biofilm, i.e. bacteria suspended in a polymer matrix, grows on a smooth wall. This measurement contains a large field of view of $1 \times 1\,\mathrm{cm}^2$. This field of view is composed of many A-scans. These A-scans are acquired by traversing the OCT-beam over the area using two galvo-mirrors. Since these two mirrors cannot occupy the same position, optical aberrations are introduced that increase with increasing distance from the center of the field of view. These aberrations manifest as a warping of the image, causing a horizontal surface to warp vertically. Most of this warping can be removed via calibration functions. In this example, we remove the remaining image warping by detecting the substratum, i.e. the surface at the bottom of the channel, and trimming any signal beneath it.

The contrast between the biofilm and the surrounding medium is much weaker than in the previous example. It is in fact so weak, that the histogram of the intensities reveals a monomodal distribution. Therefore, classical image thresholding methods, such as Otsu's method, are no longer able to separate the biofilm from the background signal. Here, we use a thresholding function that considers the shape of the distribution of intensities in a typical OCT scan, where these conditions are given. Finally, we calculate the thickness of the biofilm in one part of the scan as an example of one property that may be derived from such a scan.

1.3 Example 3 – Particle Detection

This example is used in Section 3.1.4. Since OCT enables the capture of vertical image slices through a channel, it lends itself well to particle-based flow measurements. Here, we show an example, where particles group in certain regions of the flow. However, the same evaluation procedure may be used to obtain images for velocimetry methods like PIV or PTV. Due to the low particle fraction, we again need to use the OCT-adapted thresholding.

1.4 Example 4 – Doppler-OCT in 1D

This example is used in Section 3.2.1. In this example, we show how to convert the Doppler-phase measurements into velocity profiles and compare the result to the analytical solution of a laminar channel flow. We also show how it may be necessary to scale the resulting velocities based on a known quantity, such as the flow rate or a known centerline velocity.

[a] FLOW and Fluid Physics Laboratory, Dept. of Engineering Mechanics, KTH, Stockholm SE-100 44, Sweden
[b] FLOW and SeRC (Swedish e-Science Research Centre), Dept. of Engineering Mechanics, KTH, Stockholm SE-100 44, Sweden
‡ KA and CW had equal contributions.
(Email address for correspondence: kasraa@kth.se / wittig@kth.se / shervinb@kth.se)

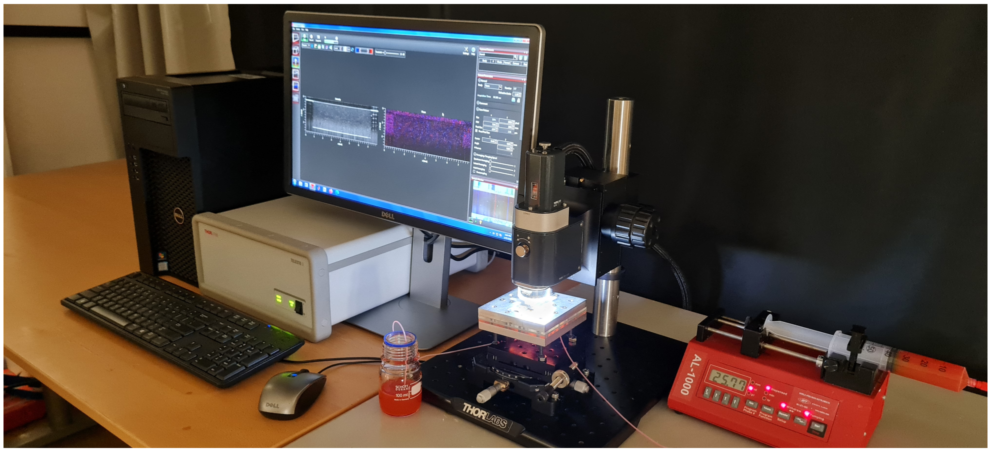

Supplementary Figure 1. The Telesto II Sd-OCT apparatus used for most measurements presented in the current manuscript.

1.5 Example 5 – Doppler-OCT in 2D

This example is used in Section 3.2.2. Doppler-OCT measurements may also be acquired along two-dimensional B-scans. Here, we use a set of B-scans to calculate the velocity over the cross-section of a channel. We calculate the evolution of the mean velocity at several locations with increasing sampling time. In this case, our results indicate that at least 250 B-scans are required to obtain a meaningful velocity field. This is caused by the high noise levels in Doppler-OCT measurements, which result in a low precision without degrading the accuracy of the results. Given this data, it is also possible to obtain reliable shear-rates even close to the wall, highlighting one of the advantages of Doppler-OCT measurements. Additionally, this data is used to demonstrate how the wall location may be detected via a maximum in the fluctuations of the intensity measurements (see Section 3.1.3).

2 System specifications

As the present manuscript is written in a device-agnostic manner, without any emphasis on the specifics of the OCT apparatus used, with all the accompanying codes solely compatible with a stack of .tiff images, throughout the paper no details and specifications of the used devices were mentioned. However, the data in this tutorial review was acquired using several OCT systems. Here, we present the specifications and limitations of these systems. The technical specifications are listed in Supplementary Table 1.

2.1 Thorlabs Telesto II

Most measurements in this tutorial review were conducted with a Spectral Domain Optical Coherence Tomography (Sd-OCT) Telesto II of Thorlabs. The central wavelength of 1310 nm and bandwidth of 270 nm are the nominal specifications of the apparatus. The spatial resolution, therefore, is approximately 2.58 μm in depth direction for water. A sample setup as used in e.g. Section 3.1.1 is shown in Supplementary Figure 1. A channel is mounted directly below the OCT probe head.

2.2 Thorlabs Ganymede GAN610-SP5

The biofilm measurement in code example 2 was acquired using a Thorlabs Ganymede GAN610-SP5 with a central wavelength of 930 nm and an LSM04 objective lens. In this configuration, an axial resolution of 4.5 μm and a lateral resolution of 12 μm can be achieved. The wavelength around 900 nm allows for measurements through several millimeters of water. Supplementary Figure 2 shows the corresponding experimental setup. Here, the probe head is mounted to a traverse that is used to move the head to several predefined measurement locations.

3 Video

Complementary to Section 4.6, a video showing a drop of lubricant (hexadecane) confined in the grooves of a millifluidic channel is provided at this link (`https://www.dropbox.com/scl/fi/y20gqt6jkekvrt49futem/Drop.avi?rlkey=az8exvd3msgttv1bar35d93ad&st=06i3e30r&dl=0`). The video has been accelerated by a factor of 30 with respect to real time.

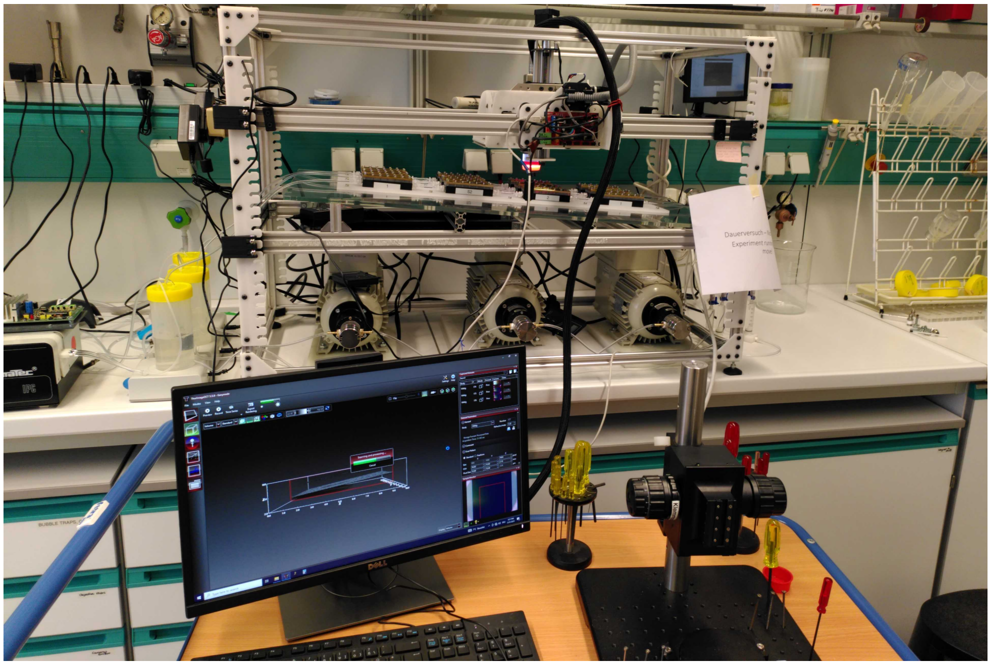

Supplementary Figure 2. The Ganymede Sd-OCT apparatus used to acquire the biofilm sample in section 3.1.2. The probe head is mounted to a traverse.
